# Organic zinc supplementation in early-lactation dairy cows and its effects on zinc content and distribution in milk and cheese

**DOI:** 10.3168/jdsc.2020-0070

**Published:** 2021-04-05

**Authors:** N.N. Xu, D.T. Yang, C. Miao, T.G. Valencak, J.X. Liu, D.X. Ren

**Affiliations:** Institute of Dairy Science, Ministry of Education Key Laboratory of Molecular Animal Nutrition, College of Animal Sciences, Zhejiang University, Hangzhou 310058, P.R. China

## Abstract

This study investigated the effects of organic zinc (Zn) supplementation in early-lactation dairy cows on Zn content and distribution in raw milk and mozzarella cheese. Thirty-four multiparous dairy cows in early lactation were randomly assigned to 2 groups: basal diet (control; CON) and basal diet supplemented with a Zn AA complex (CZ). After feeding the diets for 8 wk, raw milk was collected for manufacturing mozzarella cheese. Total Zn content and Zn distribution in raw milk and cheese were determined by atomic absorption spectrophotometry. Results showed that milk fat content was significantly increased in the CZ group compared with the CON group. No significant differences in fat, protein, and moisture contents of cheese were observed between the 2 groups. Zinc contents in milk (4.25 vs. 3.85 mg/L) and cheese (38.65 vs. 27.20 mg/kg) were significantly higher in the CZ group than in the CON group. Little Zn was lost in stretch water (<0.04 mg/L) and brine (<0.01 mg/L) during the cheese-making process. Most of the Zn was detected in casein (87.3% vs. 88.4%) of the raw milk, and then in the whey (10.6% vs. 9.88%) and fat (2.07% vs. 1.77%). Organic Zn supplementation in early-lactation cows increased Zn content in raw milk and mozzarella cheese, and Zn remained stable during cheese making.

## Highlights

• Organic Zn supplementation improves fat content in raw milk.
• Organic Zn supplementation in feed improves zinc content in cheese.
• Zinc mostly distributed in casein.
• Zinc is stable during cheese making and storage.

## Graphical Abstract

**Figure:**
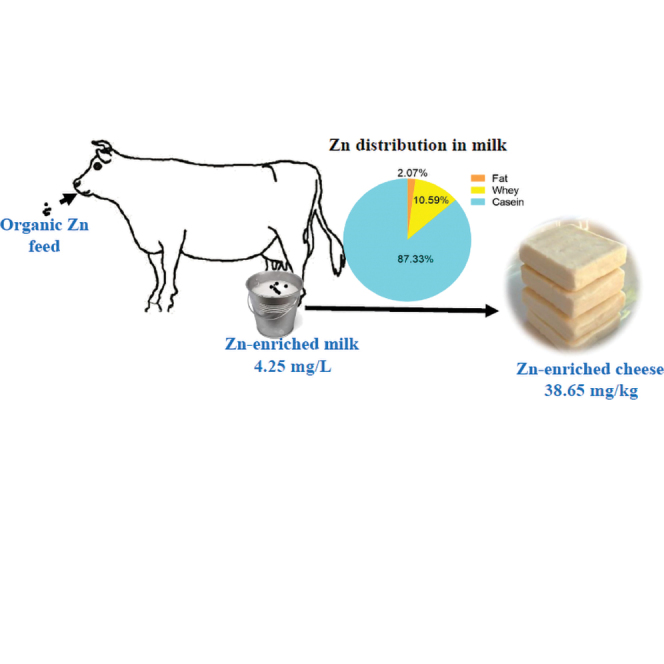
**Summary:** Zinc (Zn) is an important dietary ingredient for human and animal health. Our previous study found that feeding organic Zn to dairy cows increased Zn content in milk. In this study, supplementation of organic Zn significantly increased Zn content in both raw milk and mozzarella cheese. Most Zn was retained in cheese during cheese making. The results indicate that feeding organic Zn is beneficial for increasing Zn content in milk and cheese and that most Zn remained in cheese during cheese making and subsequent ripening processes.

Zinc (Zn) is an essential mineral for the catalytic activities of more than 100 enzymes and plays an important role in immune function, protein synthesis, and cell division. The primary cause of Zn deficiency is low dietary intake combined with limited capacity to store Zn in the body. Thus, continuous dietary intake of Zn is necessary for normal health and maintenance. However, approximately 2 billion people around the world have insufficient daily Zn intake (Song et al., 2010). Globally, almost 16 million people may suffer from Zn deficiency (Brown et al., 2004). Insufficient Zn intake restricts prepubertal physical growth of children and increases their risk of infection. Maintaining an adequate dietary Zn intake is an efficient way to combat Zn deficiency, especially among children and older adults. Recommended dietary allowances of Zn in the United States are 11 and 8 mg/d for men and women, respectively (Food and Nutrition Board and Institute of Medicine, 2001), and requirements are even higher for pregnant and nursing women.

Low dietary intake of absorbable Zn is the primary cause of Zn deficiency. In a study by Talsma et al. (2017), consuming a combination of milk and phytate-rich rice increased the total amount of absorbable Zn from rice, suggesting that Zn-rich dairy products can potentially increase dietary absorbable Zn concentration. Ianni et al. (2019) found that feeding dairy animals with Zn was a promising method to increase Zn content in dairy products. Studies have also found that Zn supplementation increases Zn content in milk (Martino et al., 2019) and that nutrition components and aromatic compound profiles of cheese can be enhanced by feeding dairy animals with Zn additives (Ianni et al., 2019; Martino et al., 2019).

Our previous study found that feeding a Zn AA complex supplement significantly improved milk protein and fat contents (C. Miao, unpublished data). Today's consumers expect high quality and health benefits from their foods (Annunziata and Mariani, 2018). Thus, high-quality cheese production should include Zn-enriched dairy products. However, the effects of feed Zn supplementation on Zn content and distribution in milk and cheese remain unclear. The aim of our study was to investigate the effects of feed Zn supplementation on Zn content and distribution in milk and mozzarella cheese as well as Zn stability during cheese storage.

Our current study was conducted at Zheng-Xing Dairy Farm, Hangzhou, China. Thirty-four healthy multiparous dairy cows in early lactation were randomly allocated into 2 groups: basal diet (control; **CON**) and basal diet supplemented with 10.4 g of a Zn AA complex (Zinpro-Zn170, Zinpro Animal Nutrition Inc.) per day per cow according to the recommended dosage (**CZ**). Zinc content in the CON and CZ groups was 60 and 140 mg/kg, respectively. The Zn AA complex product was chelated by Zn-lysine and Zn-glutamic acid 1:1 with 170 g/kg Zn in the premix. The composition and chemical profiles of the basal diet are shown in Table 1. Raw milk samples from the 2 groups were collected after 8 wk of supplemented feeding, and milk compositions were analyzed by infrared spectroscopy using a MilkoScan FT 120 (Foss Electric). Milk samples were stored at −20°C until subsequent Zn analyses.

**Table 1:** Ingredients and composition (% of DM unless otherwise noted) of the basal diet used in this study

Item	Value
Ingredient	
Spanish alfalfa	4.6
American alfalfa	8.2
Chinese oat grass	4.5
Imported oatmeal	3.4
Corn silage	21.0
Cottonseed	5.8
Beet pulp	7.0
Brewers spent grains	11.5
Concentrate	33.0
Total	100.0
Chemical profile	
CP	17.0
NDF	30.2
ADF	18.3
Ash	7.37
Ether extract	5.1
Zn, mg/kg	70.5

Thirty liters of raw milk was collected from both the CON and CZ groups at the same time and was standardized through partial skimming based on a protein:fat ratio of 3.3:3.5. Subsequently, the raw milks were pasteurized at 63°C for 30 min, followed by cooling to 35°C. Mozzarella cheese was manufactured according to the modified method as described previously (Liu et al., 2018). Cheese samples were collected after 1, 4, and 8 wk of storage. The whey, stretching water, and brine produced during cheese making were also collected and stored at −20°C until Zn content was determined. Raw milk was collected 3 times during the 9 wk, and mozzarella cheese was made upon collection. Composition of cheese samples was measured in triplicate for each batch.

The moisture, fat, and protein contents of cheese from both groups were determined with the oven-drying method (IDF, 1958), Gerber method (NSAI, 1955), and micro-Kjeldahl method (IDF, 1993), respectively. Then, the moisture content in nonfat substances and fat content in DM were calculated based on the results from basic fat and moisture contents.

The Zn content in raw milk, cheese, and by-products was analyzed according to the China national food safety standard GB 5009.14-2017 (NHFPC, 2017). Briefly, cheese (2.51 ± 0.14 g), whey (3.15 ± 0.06 mL), raw milk (4.19 ± 0.03 mL), stretch water (4.21 ± 0.05 mL), and brine (4.18 ± 0.04 mL) were initially treated with 10 mL of nitric acid and 0.5 mL of perchloric acid overnight and further digested using a microwave oven according to a digestion protocol (120°C for 60 min, 180°C for 120 min, and held at 200–220°C). Then, the ash was dissolved with 25 mL of deionized water. The Zn content of milk, cheese, and by-products was determined using atomic absorption spectrophotometry (Unicam Solaar 989, Thermo Elemental Corp.) at 213.9 nm. Zinc analysis standard curves at 0, 0.10, 0.20, 0.40, 0.80, and 1.00 mg/L Zn concentrations were prepared in 100-mL volumetric flasks with nitric acid (5%) matrix modifier from a 10.0 mg/kg Zn solution. The final Zn content was calculated according to the standard curve.

The Mixed procedure of SAS 9.4 (SAS Institute Inc.) was applied to analyze the Zn content of cheese and milk. The model used was

$$Y_{ij}=\mu +T_{i}+e_{i},$$

where *Y_ij_* was the observation of dependent variable *i*; *μ* was the population mean; *T_i_* was the effect of Zn AA complex supplementation on cheese composition and Zn content; and *e_i_* was the random error. Significance was set at *P* ≤ 0.05.

Raw milk composition is shown in Table 2. Dietary Zn supplementation significantly increased milk fat content (3.52 vs. 3.99 g/kg; *P* < 0.05). No effect was observed on milk protein content (3.32 vs. 3.38 g/kg), resulting in a lower protein:fat ratio in the CZ group (0.94 vs. 0.84; *P* < 0.05). Nayeri et al. (2014) found that dietary supplementation with a mixture of Zn sulfate and Zn AA complex was beneficial for lactation performance and milk composition. However, other studies found that dietary Zn supplementation had no effect on raw milk and cheese composition (Cope et al., 2009; Ianni et al., 2019). Milk content was not sensitive to Zn supplementation based on ZnO and Zn sulfate (Cope et al., 2009; Wang et al., 2013). Similarly, our current study did not reveal a significant improvement in milk yield in the CZ group (data not shown). The mechanism remains unclear. We hypothesize that both the biochemical nature and the dosage of Zn might play key roles in improving milk yield and composition.

**Table 2:** Composition of raw milk and cheese (means ± SD) from the 2 experimental groups^1^

Item	CON	CZ
Raw milk		
Protein, g/kg	3.32 ± 0.01	3.38 ± 0.01
Fat, g/kg	3.52 ± 0.02^b^	3.99 ± 0.01^a^
Protein/fat	0.94 ± 0.00^a^	0.84 ± 0.01^b^
Cheese, %		
Fat	21.34 ± 1.65	22.02 ± 0.5
Protein	19.64 ± 0.65	20.11 ± 0.15
Moisture	52.96 ± 1.51	50.50 ± 0.62
Fat in DM	45.37 ± 3.16	44.48 ± 1.10
MNFS^2^	67.32 ± 1.79	66.76 ± 0.86

a,b Means within a row with different superscripts were significantly different (*P* < 0.05).

1 CON = basal diet (control; n = 3); CZ = basal diet supplemented with 10.4 g of a Zn AA complex (Zinpro-Zn170, Zinpro Animal Nutrition Inc.) per day per cow according to the recommended dosage (n = 3).

2 Moisture in nonfat substances.

The composition of cheese is shown in Table 2. No differences in fat, protein, and moisture contents, fat content in DM, and moisture content in nonfat substances were observed between CON and CZ cheeses, probably because the milk had to undergo a standardization process before manufacturing cheese. Ianni et al. (2019) also found that Zn supplementation in Friesian cows did not affect fat and protein contents in fresh and ripened cheese. Martino et al. (2019) found that the composition of fresh and ripened cheese did not differ between the control and the Zn-supplemented group. Results of our study indicate that dietary Zn supplementation as in the CZ group did not affect any of the cheese micronutrients and moisture content.

The Zn content of milk is shown in Table 3. The highest recommended dietary Zn content for dairy cows is 73 mg/kg of DM over the whole life cycle (NRC, 2001), indicating that the supplemented Zn content in our current study was sufficient. The Zn content of CON milk was comparable with that of raw milk in a study reported by Raynal-Ljutovac et al. (2008). The additional dietary Zn supplementation increased the Zn content (3.85 vs. 4.25 mg/L) of milk (*P* < 0.05) as expected. Similarly, Martino et al. (2019) found that a Zn-enriched diet significantly increased Zn content of milk. However, no effect was reported on cheese samples. As for Zn distribution in milk, no differences in whey, casein, and fat were observed between the 2 groups (Table 3). Hence, organic dietary Zn supplementation did not affect Zn distribution in milk. The Zn import and export metabolic process is strictly regulated by the mammary gland to maintain sufficient Zn supply to the neonate calf (Kelleher and Lönnerdal, 2005). This mechanism could explain the slight improvement in Zn concentration following supplementation.

**Table 3:** Zinc concentration (means ± SD) and distribution in different components of raw milk and cheese^1^

Item	CON	CZ
Zinc in raw milk		
Total, mg/L	3.85 ± 0.07^b^	4.25 ± 0.07^a^
Casein, %	88.35 ± 1.24	87.33 ± 2.08
Whey, %	9.88 ± 0.78	10.59 ± 1.06
Fat, %	1.77 ± 0.18	2.07 ± 0.13
Zinc in cheese		
Total, mg/kg	27.20 ± 1.56^b^	38.65 ± 3.18^a^
Whey, mg/kg	0.18 ± 0.03	0.27 ± 0.09
Stretch water, mg/L	<0.02	<0.04
Brine, mg/L	ND^2^	ND

a,b Means within a row with different superscripts were significantly different (*P* < 0.05).

1 CON = basal diet (control; n = 3); CZ = basal diet supplemented with 10.4 g of a Zn AA complex (Zinpro-Zn170, Zinpro Animal Nutrition Inc.) per day per cow according to the recommended dosage (n = 3).

2 Not detected.

Most Zn in milk is bound to the casein protein, and thus Zn enrichment is expected during the cheese-making process. In mozzarella cheese, the Zn content at normal Zn supply is 31 mg/kg (USDA, 2010). Dietary Zn supplementation significantly (*P* < 0.05) increased the Zn content of cheese (27.20 vs. 38.65; *P* < 0.05). A higher Zn concentration in whey was observed in the CZ group (0.18 vs. 0.27), which probably was due to the higher Zn distribution in milk whey (Table 3). Little Zn was observed in stretch water (<0.04 mg/L) and brine (<0.01 mg/L) in both cheeses, indicating that Zn was not water soluble during cheese making. Because 85% of the Zn in milk was detected in casein micelles (Table 3), more than 95% of Zn in milk was maintained in the cheeses of both groups during the 8-wk storage period. Another study by Singh et al. (1989) showed that almost 100% of Zn was retained in cheese. Furthermore, Zn remained stable during cheese storage for 2 mo, as almost all Zn was retained (98%). Thus, dietary Zn supplementation provides a potential way to produce Zn-enriched milk. Similarly, Zn enrichment may have beneficial effects on human health through improved dairy products such as mozzarella cheese.

Sufficient daily Zn intake is essential for human health. To maintain physiological levels despite deficient Zn storage capabilities, increased Zn concentrations in raw milk may provide a suitable source of Zn for humans, especially those who do not consume enough meat. Coudray (2011) found that milk products represent a key source of dietary Zn in children. It was also reported that a significant association existed between intake of dairy products and serum Zn levels in children (Schlegel-Zawadzka et al., 2002). The increased intake of Zn from dairy products can further be improved by Zn absorption from rice, resulting in higher net daily Zn consumption. Thus, the significantly higher Zn concentration in milk and cheese indicated that dietary Zn supplementation in early lactation provides a useful method for Zn enrichment of dairy products. This may serve body health, and the consumption of Zn-enriched cheese may become a convenient and efficient way to combat potential Zn deficiency.

In conclusion, dietary supplementation of organic Zn at 80 mg/kg improved milk fat content. No difference in mozzarella cheese protein and fat contents was observed between the CON and CZ groups. Zinc content in raw milk was significantly improved by dietary Zn supplementation, and almost 90% of Zn was distributed in casein. The remaining Zn was found in cheese during cheese making, and Zn remained stable during storage. Our study indicates that dietary Zn supplementation improves the Zn content in both raw milk and cheese. These findings may be useful for the development of functional, nutritious dairy products.
