# Erratum to “Organic zinc supplementation in early-lactation dairy cows and its effects on zinc content and distribution in milk and cheese” (JDS Commun. 2:110–113)

**DOI:** 10.3168/jdsc.2022-3-2-166

**Published:** 2022-03-16

**Authors:** N.N. Xu, D.T. Yang, C. Miao, T.G. Valencak, J.X. Liu, D.X. Ren

The animal ethics statement was missing from this paper. The following statement should be included: “The animal use protocol listed in this paper was reviewed and ethical approval for the study was given by the Laboratory Animal Center of Zhejiang University.”
